# Dietary intake of vitamins A, B, C, D and E and risk of islet autoimmunity and type 1 diabetes in genetically at-risk children: a prospective study from the DIPP birth cohort

**DOI:** 10.1007/s00125-025-06635-9

**Published:** 2025-12-19

**Authors:** Markus Mattila, Peppi Haario, Leena Hakola, Hanna-Mari Takkinen, Essi J. Peltonen, Tuuli E. Korhonen, Suvi Ahonen, Jorma Ilonen, Jorma Toppari, Mikael Knip, Riitta Veijola, Sari Niinistö, Suvi M. Virtanen

**Affiliations:** 1 Faculty of Social Sciences, Unit of Health Sciences, Tampere University, Tampere, Finland; 2 Tampere University Hospital, Wellbeing Services County of Pirkanmaa, Tampere, Finland; 3 Department of Public Health, Finnish Institute for Health and Welfare, Helsinki, Finland; 4 Immunogenetics Laboratory, Institute of Biomedicine, University of Turku, Turku, Finland; 5 Research Centre for Integrative Physiology and Pharmacology, Institute of Biomedicine, University of Turku, Turku, Finland; 6 Centre for Population Health Research, University of Turku and Turku University Hospital, Turku, Finland; 7 InFLAMES Research Flagship Center, University of Turku, Turku, Finland; 8 Turku University Hospital, Department of Pediatrics, Turku, Finland; 9 Research Program for Clinical and Molecular Metabolism, Faculty of Medicine, University of Helsinki, Helsinki, Finland; 10 Tampere University Hospital, Department of Pediatrics, Tampere, Finland; 11 Department of Pediatrics, PEDEGO Research Unit, Medical Research Center, University of Oulu, Oulu, Finland; 12 Oulu University Hospital, Department of Children and Adolescents, Oulu, Finland; 13 Centre for Child, Adolescent and Maternal Health Research, Tampere University and Tampere University Hospital, Tampere, Finland

**Keywords:** Birth cohort study, Child, Diabetes mellitus type 1, Islet autoimmunity, Joint models, Tocopherols, Vitamin A, Vitamin B complex, Vitamin C, Vitamin D, Vitamin E

## Abstract

**Aims/hypothesis:**

In this prospective birth cohort study, we examined whether the dietary intake of A, B, C, D and E vitamins is associated with the risk of islet autoimmunity or type 1 diabetes in children who are genetically at risk for type 1 diabetes.

**Methods:**

Data on vitamin intakes in the Finnish Type 1 Diabetes Prediction and Prevention (DIPP) cohort study were available for 5674 children born between September 1996 and September 2004 in Oulu University Hospital or Tampere University Hospital. Diet was assessed using 3-day food records at the age of 3 and 6 months, and annually from 1 to 6 years. The primary outcomes were: (1) islet autoimmunity defined as repeated positivity for islet cell autoantibodies and at least one out of three type 1 diabetes-related biochemical autoantibodies or a diagnosis of type 1 diabetes; and (2) a diagnosis of type 1 diabetes.

**Results:**

During the 6-year follow-up, 247 children (4.4%) developed islet autoimmunity and 94 (1.7%) developed type 1 diabetes. When adjusted for total energy intake, sex, HLA genotype and family history of diabetes, the intakes of retinol (HR 0.91; 95% credible interval [CrI] 0.86, 0.97 per 10 µg/MJ increase in intake), vitamin C (HR 0.70; 95% CrI 0.54, 0.90 per 10 µg/MJ) and vitamin E (HR 0.93; 95% CrI 0.89, 0.97 per 0.1 mg/MJ) were associated with decreased risk of islet autoimmunity and also with the risk of type 1 diabetes (retinol: HR 0.83; 95% CrI 0.74, 0.96 per 10 µg/MJ; vitamin C: HR 0.45; 95% CrI 0.23, 0.84 per 10 µg/MJ; vitamin E: HR 0.89; 95% CrI 0.80, 0.99 per 0.1 mg/MJ). The associations remained statistically significant after multiple testing correction for the risk of islet autoimmunity but not for type 1 diabetes. The association between retinol intake and islet autoimmunity risk was not significant when the 3-month age point, during which the child is primarily breastfed, was excluded. We observed a weak inverse association for vitamin D and islet autoimmunity, and no associations for B vitamins.

**Conclusions/interpretation:**

High intake of vitamin C and vitamin E was associated with a decreased risk of islet autoimmunity.

**Graphical Abstract:**

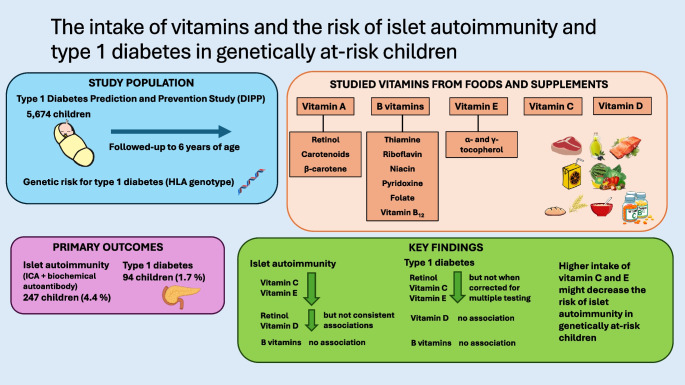

**Supplementary Information:**

The online version of this article (10.1007/s00125-025-06635-9) contains peer-reviewed but unedited supplementary material.


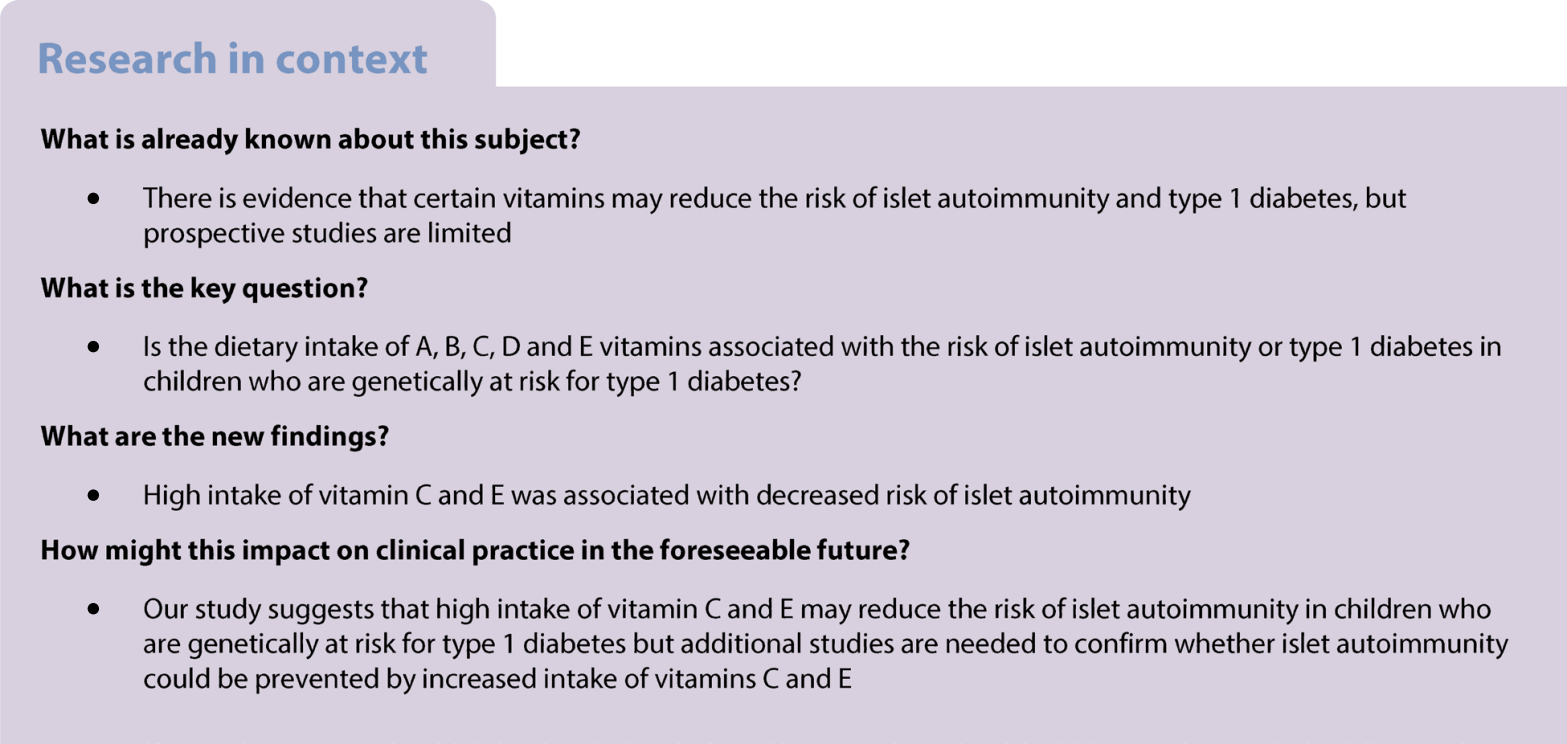


## Introduction

Environmental factors such as dietary factors have been suggested to play a significant role in islet autoimmunity (IA) and type 1 diabetes development [1]. Dietary factors may act either as triggers, promotors or inhibitors of autoimmune reactions, and thus affect the development of type 1 diabetes [1–3]. Vitamins represent a heterogenous group of organic molecules and essential micronutrients in the diet that are required for several metabolic functions. Vitamins may play a role in type 1 diabetes development, but studies assessing the association between the intake of vitamins during early childhood and the risk of IA or type 1 diabetes are few [2, 3]. Vitamin D is of interest due to its role in the regulation of the immune system. In prospective studies, high plasma/serum vitamin D status in childhood was associated with decreased risk of IA in some studies [4, 5], but no association was found in the Finnish Type 1 Diabetes Prediction and Prevention (DIPP) study [6, 7]. Use of vitamin D supplements was associated with a lower risk of type 1 diabetes in a Northern Finland birth cohort [8]. Vitamins A, C and E are dietary antioxidants that could protect against the development of autoimmunity by suppressing the accumulation of oxygen radicals. Retrospective case–control studies have suggested that high blood vitamin A levels in the form of retinol and retinol-binding protein status could protect against the development of type 1 diabetes [9, 10]. An animal study suggested that a diet fortified with vitamin A (retinyl acetate) could prevent the development of type 1 diabetes [11], but there are no such studies in humans. High plasma ascorbic acid (vitamin C) in childhood was associated with a decreased risk of IA in the multinational prospective TEDDY study (The Environmental Determinants of Diabetes in the Young) [12]. Furthermore, a retrospective case–control study suggested that the use of a vitamin C supplement in childhood is associated with a decreased risk of type 1 diabetes [13]. Prospective studies have provided some evidence that high plasma vitamin E status is associated with decreased or borderline decreased risk of type 1 diabetes [14, 15] but another study found no association with IA risk [16]. B vitamins act as essential cofactors in numerous cellular reactions [17, 18] such as immune functions [18]. Studies on the role of B vitamins in the risk of IA or type 1 diabetes are few and inconsistent. In a recent study in the TEDDY cohort, high intake of niacin, pyridoxine and vitamin B_12_ was associated with decreased risk of IA, but intake of riboflavin was associated with increased risk of IA [19]. Another study in the DAISY cohort (Diabetes Autoimmunity Study in the Young) assessing metabolomics-related nutrient patterns suggested that a pattern comprising high intake of riboflavin, niacin and vitamin B_12_ could decrease the risk of progression from IA to clinical type 1 diabetes [20]. However, large prospective studies on dietary intake of vitamins and risk of IA or type 1 diabetes are still limited.

The main aim of the current prospective birth cohort study was to explore whether intake of vitamins A, B, C, D and E from foods and supplements is associated with the development of IA or type 1 diabetes in children who are genetically at risk for type 1 diabetes.

## Methods

### Participants

**Fig. 1:**
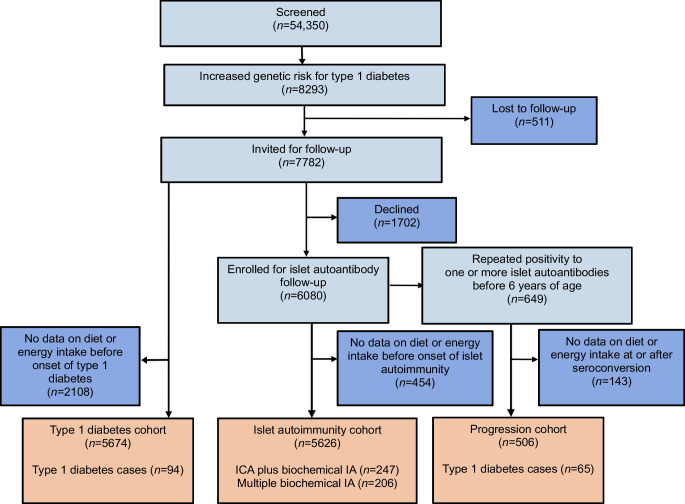
DIPP study participant flow chart. All participants in the IA cohort are within the type 1 diabetes genetic risk cohort, and all participants in the progression cohort are within the IA cohort

This study is a part of the Nutrition Study within the larger DIPP study, which is an ongoing multidisciplinary prospective population-based birth cohort study screening for *HLA-DQB1*-conferred predisposition to type 1 diabetes using cord blood samples (ClinicalTrials.gov NCT03269084) [21]. Families with newborn infants born between September 1996 and September 2004 were recruited from the regions served by Oulu University Hospital and Tampere University Hospital in Finland. Children with high or moderate HLA-conferred risk (approximately 14% of the whole population) were eligible for the study and were invited to participate. The exclusion criteria were any severe systemic disease or congenital anomaly, or if the child’s parents were of non-European origin or did not speak Finnish, Swedish or English fluently. Altogether 6080 children were enrolled in this follow-up study. In the present study, the 6-year follow-up period was used. The inclusion criteria for the IA cohorts were the availability of at least one autoantibody assessment and at least one completed day in a 3-day food record at or before the assessment of autoantibodies. The inclusion criteria for the type 1 diabetes genetic risk cohort were available information on the type 1 diabetes status of the child and at least one completed day in a 3-day food record at or before the diagnosis. The inclusion criteria for the progression cohort were repeated positivity for at least one autoantibody and at least one completed day in a 3-day food record at or after the first assessment of seropositivity but before type 1 diabetes diagnosis. A total of 5626 children were included in the IA cohort, 5674 in the type 1 diabetes genetic risk cohort, and 506 in the progression cohort. The DIPP Nutrition Study flow chart is presented in Fig. 1.

### Outcome variables

ICA were screened at 3–12 month intervals as described previously [22]. If a participant tested positive for ICA, all available samples from that participant were analysed for IAA, GADA and IA-2A. ICA were quantified by a standard indirect immunofluorescence method, while IAA, GADA and IA-2A were quantified using specific radiobinding assays [23]. In the current study, two outcome definitions for IA were used: (1) repeated positivity for ICA and at least one biochemical autoantibody or a diagnosis of type 1 diabetes (ICA plus biochemical IA); and (2) repeated positivity for at least two biochemical autoantibodies or a diagnosis of type 1 diabetes (multiple biochemical IA). Both IA outcomes were assessed by February 2017. The type 1 diabetes outcome was defined by a diagnosis of type 1 diabetes obtained from the Finnish Paediatric Diabetes Register and the university hospitals by May 2017. The Finnish Paediatric Diabetes Register covers approximately 92% of children diagnosed with type 1 diabetes. Children who are not identified in the diabetes register as having type 1 diabetes were considered free from type 1 diabetes. Progression from islet autoantibody positivity to type 1 diabetes was assessed among children who were repeatedly positive for at least one of the assessed autoantibodies. In the analyses, the primary outcomes were: (1) ICA plus biochemical IA; and (2) type 1 diabetes. Secondary outcomes were multiple biochemical IA and progression from IA to type 1 diabetes.

### Genetic methods

HLA-DQ genotyping using panels of sequence-specific oligonucleotide probes has been described previously [22]. The HLA-DQB1(*02/*0302) genotype represents high risk for type 1 diabetes, and the HLA-DQB1*0302/x genotype (where *x* indicates alleles other than DQB1 *02, *0301 or *0602/3) represents moderate risk. After April 1997, the DQB1*0602/3 probe recognising both DQB1*06:02 and DQB1*06:03 alleles was replaced by a probe specific for DQB1*06:02.

### Ethical aspects

The DIPP study adheres to the Declaration of Helsinki, and the local ethics committees of Oulu and Tampere University Hospitals approved the study protocol (ETL 97193M). Families gave their written informed consent for the genetic testing of the newborn infant and for their participation in the follow-up study, and were able to discontinue the study whenever they want.

### Assessment of diet

The intake of vitamins from food (including breastmilk and drinks) and dietary supplements was assessed by using 3-day food records containing two weekdays and one weekend day at the 3, 6 and 12 month visits and the 2, 3, 4, 5 and 6 year visits. The collection of food consumption data has been described in detail previously [24, 25]. The food and nutrient calculations were based on the constantly updated in-house nutrition calculation software Finessi (Finnish Institute for Health and Welfare, Finland), which utilises the Finnish National Food Composition Database (Fineli) [26]. Calculated dietary components used in the statistical analysis include energy (MJ), vitamin A, retinol, β-carotene, carotenoids, thiamine (B_1_), riboflavin (B_2_), niacin (B_3_), pyridoxine (B_6_), folate (B_9_), vitamin B_12_, vitamin C, vitamin D, vitamin E (α-tocopherol) and γ-tocopherol. All vitamins were measured as milligrams or micrograms. Vitamin A comprised retinol and carotenoids with vitamin A activity (retinol activity equivalents). Carotenoids comprised of α-, β- and γ-carotenes and β-cryptoxanthin. Information on pantothenic acid and biotin intake in our food composition data was not up to date, and therefore those B vitamins were not included in this study. For breastfed children, we estimated the total energy intake based on age, body weight and the expected energy requirements for growth and development [27]. The amount of ingested breastmilk was estimated on the basis of weight, growth rate and energy intake from other foods [28]. Vitamin values in the food composition database are based on chemical analysis of food samples, recipe calculation and adopting values from other sources, e.g. other databases, scientific literature and food labelling. Fineli recipe calculations are performed according to European Food Information Resource (EuroFIR) guidelines [29], and nutrient retention factors are based on a report from the National Food Administration, Sweden [30]. As children’s diets change and expand during growth, we analysed the food sources for vitamins in two age groups: up to 1 year of age (3, 6 and 12 months) and from 2–6 years of age. We reviewed and updated the values for fat-soluble vitamins such as vitamin A and E in the Fineli database in 2012–2016 by screening and correcting possible errors and by quality checking of the new database versions' which improved the accuracy of data on fat-soluble vitamins.

### Sociodemographic characteristics

Information on diabetes status (all types of diabetes) in first-degree relatives (yes, no), the child’s sex (male, female) and maternal education (none, vocational, secondary vocational, university studies, or degree) were collected from parents using a structured questionnaire. We were not allowed to register the children’s ethnicity due to study regulations. However, the selected participants were of European origin, as the HLA-DQ genotypes of interest may have lower predictive value in non-European populations [22]. Weight was assessed at each study visit and weight-for-age *z* scores were calculated separately for both sexes based on WHO criteria [31].

### Statistical methods

Joint models that combine longitudinal and survival data into a single model [32] were used to analyse the association between the intake of each vitamin (between 3 months to 6 years) and the development of IA and type 1 diabetes in children by the age of 6 years. The exposure was modelled using a linear mixed-effects model, and a Cox proportional hazards regression model was used to build the time-to-event submodel. Joint models are especially useful with longitudinally collected exposure data, enabling reconstruction of a complete dietary intake profile for each participant even if a series of repeated measurements is incomplete due to dropout or missed diet records. A Bayesian framework was used to estimate the posterior distribution of model parameters, providing credible intervals (CrI) that reflect the probability of the parameter lying within a given range, unlike confidence intervals that rely on repeated sampling assumptions. Model fitting was performed using Markov chain Monte Carlo (MCMC) sampling, allowing flexible evaluation of spline-based hazard functions. Three chains were run, and convergence was confirmed (Gelman–Rubin statistic <1.1). The event time for the IA outcomes was defined as the midpoint between the first repeatedly positive test and the preceding sample. The event time for the type 1 diabetes outcome was the diagnosis date. A current-value association structure was used, and thus the HR at a given point in time *t* is provided for a 1 unit (0.1, 1, 10 or 100 mg or µg/MJ) increase in the longitudinal value of the vitamin intake at the same time point *t*. Vitamin intake from 3 months to 6 years of age was modelled using piecewise natural cubic spline functions with three knots in the linear mixed-effects submodels. The locations of knots were defined using an algorithm that selected the best suitable combination of knots by fitting all relevant combinations and selecting the best-fitting model based on the Bayesian information criterion. All analyses were adjusted for variables that had previously been observed to be potential confounders: total energy intake, sex, HLA genotype (high or moderate risk) and family history of diabetes of any type [33–35]. For energy adjustment, the multivariate nutrient density method [33] was used, in which absolute vitamin intake is divided by the total energy intake in MJ, and the energy-adjusted intake as well as the total energy intake are included in the model as longitudinal covariates. We controlled for multiple testing by using the Benjamini–Hochberg procedure to calculate the false discovery rate with a significance level of 0.05 [36]. The progression analysis was performed from the time of the first seroconversion until diagnosis of type 1 diabetes or the age of 6 years, and the analyses were adjusted for the age at first seroconversion in addition to other potential confounders. The use of the joint model has been described in more detail elsewhere [37].

We performed additional analyses on the risk of ICA plus biochemical IA and type 1 diabetes outcomes adjusted for maternal education and breastfeeding status at the age of 6 months. As these adjustments did not change the results, we did not include them in the model presented. Furthermore, we performed outcome analysis adjusting for weight-for-age *z* score to assess whether growth/overweight confounds our results. As the children were primarily breastfed at 3 months of age, with limited other dietary sources, we performed a sensitivity analysis on the associations between vitamin intakes and the risk of ICA plus biochemical IA excluding the 3-month age point. To study whether the association between the vitamin intakes and the risk of ICA plus biochemical IA varied over time, the interaction term between the vitamin intake and a natural cubic spline of age with one knot at 3 years (the midpoint of food consumption assessment) was added to the model. The model was compared with the original one using the deviance and Watanabe–Akaike’s information criteria, and log pseudo-marginal likelihood [38]. If at least two of the criteria suggested the existence of a time-varying coefficient, the association was visually inspected. As the number of children with type 1 diabetes was low (*n*=94), we did not assess the interaction of time with the type 1 diabetes outcome. We also performed an interaction analysis to test whether sex or HLA genotypes modify the associations between vitamin intakes and the risks of ICA plus biochemical IA and type 1 diabetes. To assess whether food avoidance influenced the association between vitamin intake and outcomes, we performed a sensitivity analysis excluding children who avoided fruits, vegetables, dairy or cereals. We also tested whether vitamin supplement use (yes vs no) was associated with the risk of ICA plus biochemical IA using Cox regression. Differences between energy-adjusted vitamin intakes by background variables at 6 months and 2 years of age were assessed using one-factor ANOVA and unpaired *t* test. The analyses were performed using the joint model function from the JMbayes2 package in R version 4.2.1 (https://www.r-project.org/) and IBM SPSS Statistics version 29.0 (IBM).

## Results

### Descriptive characteristics

**Fig. 2:**
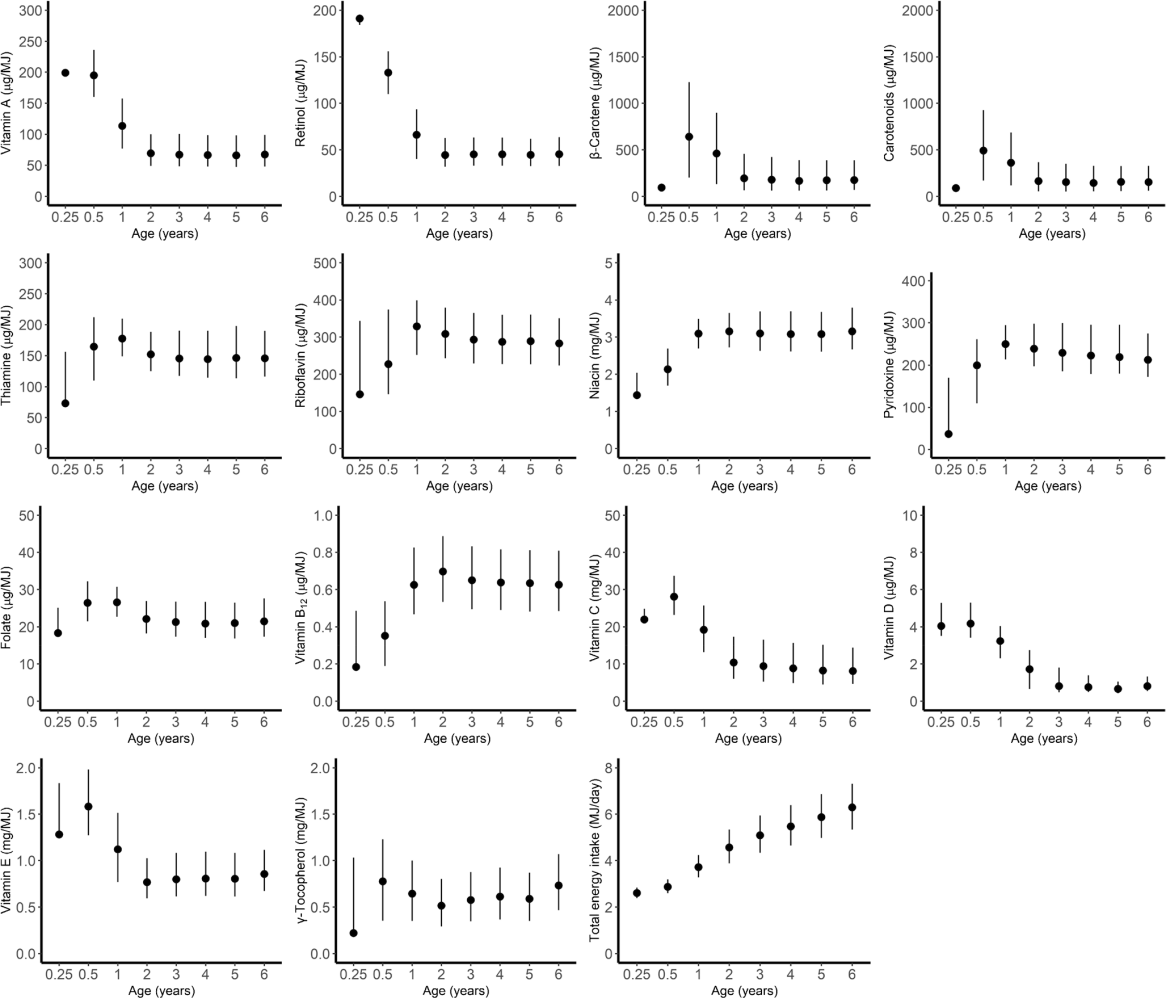
Energy-adjusted vitamin intakes and energy intake by age in 5674 children in the DIPP study. Values are medians (IQR)

During the 6 year follow-up, 247 children (4.4%) developed ICA plus biochemical IA, while 206 children (3.7%) developed multiple biochemical IA (Fig. 1). Of the 5674 children in the type 1 diabetes genetic risk cohort, 94 children (1.7%) developed type 1 diabetes during the 6-year follow-up. There were 48 more children in the diabetes genetic risk cohort than in the IA cohort, because 48 children with dietary data did not have autoantibody assessments and were classified based on information from the register. A total of 506 children developed repeated positivity for at least one of the autoantibodies (progression cohort), of whom 65 (12.8%) developed type 1 diabetes by the age of 6 years. The distributions of children by background characteristics and study outcomes are presented in Table 1. The dropout rates among the 5626 participants at 1, 2 and 6 year follow-up were 6%, 14% and 35%, respectively, for serum autoantibody measurements and 15%, 25% and 55%, respectively, for food records. The total number of food record days was 81,075 in the 5674 children aged 3 months to 6 years, and the mean number of food record days per child was 14.3. Median energy-adjusted vitamin and energy intakes by age are presented in Fig. 2. The vitamin intakes from dietary sources and dietary supplements are presented in electronic supplementary material (ESM) Results and ESM Tables 1 and 2.

**Table 1:** Characteristics of the children in the DIPP study

	IA cohort			Type 1 diabetes genetic risk cohort		Progression cohort^a^	
Characteristic	Total (*n*=5626)	ICA plus biochemical IA (*n*=247)	Multiple biochemical IA (*n*=206)	Total (*n*=5674)	Type 1 diabetes (*n*=94)	Total (*n*=506)	Type 1 diabetes (*n*=65)
Sex							
Male	2988 (53.1)	148 (59.9)	126 (61.2)	3010 (53.0)	54 (57.4)	285 (56.3)	42 (64.6)
Female	2638 (46.9)	99 (40.1)	80 (38.8)	2664 (47.0)	40 (42.6)	221 (43.7)	23 (35.4)
*HLA-DQB1*-conferred risk^b^							
High	1102 (19.6)	77 (31.2)	69 (33.5)	1109 (19.5)	35 (37.2)	116 (22.9)	23 (35.4)
Moderate	4524 (80.4)	170 (68.8)	137 (66.5)	4565 (80.5)	59 (62.8)	390 (77.1)	42 (64.6)
Family history of diabetes^c^							
Yes	333 (5.9)	30 (12.1)	29 (14.1)	333 (5.9)	14 (14.9)	43 (8.5)	9 (13.8)
No	5080 (90.3)	211 (85.4)	171 (83.0)	5125 (90.3)	78 (83.0)	453 (89.5)	55 (84.6)
Missing	213 (3.8)	6 (2.4)	6 (2.9)	216 (3.8)	2 (2.1)	10 (2.0)	1 (1.5)
Maternal education							
None	356 (6.3)	26 (10.5)	21 (10.2)	360 (6.3)	8 (8.5)	36 (7.1)	4 (6.2)
Vocational school or course	1499 (26.6)	61 (24.7)	50 (24.3)	1510 (26.6)	21 (22.3)	120 (23.7)	12 (18.5)
Secondary vocational education	2395 (42.6)	88 (35.6)	71 (34.5)	2412 (42.5)	34 (36.2)	213 (42.1)	23 (35.4)
University studies or degree	1220 (21.7)	65 (26.3)	58 (28.2)	1234 (21.7)	28 (29.8)	128 (25.3)	24 (36.9)
Missing	156 (2.8)	7 (2.8)	6 (2.9)	158 (2.8)	3 (3.2)	9 (1.8)	2 (3.1)
Breastfed at 6 months of age							
Yes	3254 (57.8)	132 (53.4)	107 (51.9)	3262 (57.5)	45 (47.9)	288 (56.9)	37 (56.9)
No	2130 (37.9)	106 (42.9)	89 (43.2)	2141 (37.7)	42 (44.7)	211 (41.7)	27 (41.5)
Missing	242 (4.3)	9 (3.6)	10 (4.9)	271 (4.8)	7 (7.4)	7 (1.4)	1 (1.5)
Age at seroconversion (years)		2.5 (1.3–3.6)^d^	2.5 (1.3–3.6)^d^			1.9 (1.2–3.5)^e^	
Age at type 1 diabetes diagnosis (years)					4.0 (2.9–5.0)		4.2 (3.0–5.0)

Values are *n* (%) for categorical variables and median (IQR) for continuous variables

^a^The criteria for inclusion in the progression cohort were repeated positivity for at least one autoantibody and availability of at least one food record after seroconversion. Based on these criteria, 65 out of 94 children who developed type 1 diabetes by the age of 6 years were included

^b^High-risk genotype: human leukocyte antigen-DQB1 isotype (*HLA-DQB1*)(*02/*03:02). Moderate-risk genotype HLA-DQB1*03:02/x (x ≠*02, *03:01, *06:02)

^c^All types of diabetes

^d^Seroconversion age at which the child has repeated positivity for islet cell autoantibodies or any of the biochemical autoantibodies

^e^Initial seroconversion age at which the child has first positivity for any of the biochemical autoantibodies

### Associations between vitamins and type 1 diabetes-related outcomes

The intakes of vitamin A, retinol, vitamin C and vitamin E were associated with decreased risk of both IA outcomes, while intake of vitamin D was associated with decreased risk of ICA plus biochemical IA (Table 2). The intakes of retinol, β-carotene, vitamin C and vitamin E were associated with decreased risk of type 1 diabetes (Table 2). When corrected for multiple testing, the associations were significant for the intakes of retinol, vitamin C and vitamin E with both IA outcomes (Table 2). Further adjustment for weight-for-age *z* score did not change the associations between vitamin intakes and ICA plus biochemical IA (data not shown).

**Table 2:** Child’s intake of A, B, C, D and E vitamins and the risk of IA, type 1 diabetes and progression from IA to type 1 diabetes at the age of 3 months to 6 years

	ICA plus biochemical IA cohort			Multiple biochemical IA cohort			Type 1 diabetes genetic risk cohort			Progression cohort	
	HR (95% CrI)^a^ (*n*=5626 participants, *n*=247)	*p* value^b^	*p* value^c^	HR (95% CrI)^a^ (*n*=5626 participants, *n*=206)	*p* value^b^	*p* value^c^	HR (95% CrI)^a^ (*n*=5674 participants, *n*=94)	*p* value^b^	*p* value^c^	HR (95% CrI)^d^ (*n*=506 participants, *n*=65)	*p* value^b^
Vitamin A (10 µg/MJ)	0.95 (0.89, 0.99)	0.019	0.106	0.84 (0.79, 0.89)	<0.001	0.003	0.97 (0.86, 1.04)	0.589		0.90 (0.79, 1.02)	0.122
Retinol (10 µg/MJ)	0.91 (0.86, 0.97)	0.003	0.034	0.78 (0.74, 0.84)	<0.001	0.003	0.83 (0.74, 0.96)	0.013	0.080	0.92 (0.79, 1.04)	0.267
β-Carotene (100 µg/MJ)	0.97 (0.90, 1.03)	0.379		0.94 (0.86, 1.01)	0.100		0.84 (0.69, 1.00)	0.049	0.211	0.93 (0.64, 1.24)	0.692
Carotenoids (100 µg/MJ)	0.98 (0.93, 1.02)	0.398		0.95 (0.89, 1.01)	0.095		0.88 (0.77, 1.01)	0.071		0.98 (0.78, 1.20)	0.872
B vitamins											
Thiamine (10 µg/MJ)	0.99 (0.96, 1.01)	0.455		0.99 (0.95, 1.01)	0.673		0.98 (0.93, 1.01)	0.290		1.03 (0.95, 1.10)	0.455
Riboflavin (10 µg/MJ)	1.00 (0.99, 1.00)	0.967		1.00 (0.99, 1.01)	0.710		1.00 (0.97, 1.01)	0.848		1.00 (0.95, 1.05)	0.976
Niacin (1 mg/MJ)	1.08 (0.88, 1.30)	0.459		1.19 (0.97, 1.43)	0.100		1.05 (0.66, 1.49)	0.749		1.05 (0.54, 1.89)	0.833
Pyridoxine (10 µg/MJ)	1.00 (0.99, 1.01)	0.767		1.00 (0.99, 1.01)	0.448		0.99 (0.96, 1,01)	0.693		1.03 (0.95, 1.10)	0.455
Folate (10 µg/MJ)	0.96 (0.72, 1.20)	0.802		1.01 (0.74, 1.26)	0.854		0.91 (0.46, 1.31)	0.841		1.63 (0.88, 2.69)	0.105
Vitamin B_12_ (0.1 µg/MJ)	1.02 (0.96, 1.06)	0.403		1.03 (0.96, 1.07)	0.342		1.00 (0.85, 1.08)	0.761		0.96 (0.73, 1.14)	0.844
Vitamin C (10 mg/MJ)	0.70 (0.54, 0.90)	0.005	0.047	0.68 (0.51, 0.89)	0.006	0.048	0.45 (0.23, 0.84)	0.009	0.063	0.92 (0.48, 1.66)	0.825
Vitamin D (1 µg/MJ)	0.86 (0.74, 0.99)	0.031	0.145	0.88 (0.75, 1.01)	0.084		0.93 (0.78, 1.03)	0.395		1.13 (0.74, 1.71)	0.553
Vitamin E (0.1 mg/MJ)	0.93 (0.89, 0.97)	0.001	0.014	0.92 (0.87, 0.97)	<0.001	0.003	0.89 (0.80, 0.99)	0.026	0.132	0.97 (0.87, 1.06)	0.620
γ-Tocopherol (0.1 mg/MJ)	0.97 (0.92, 1.02)	0.317		1.00 (0.94, 1.05)	0.894		0.98 (0.84, 1.10)	0.875		0.98 (0.85, 1.12)	0.845

^a^HR and CrIs from joint models per 1 unit increase in intake, e.g. per 10 µg/MJ increase for vitamin A. Adjusted for sex, HLA genotype, family history of diabetes of any type, and total energy intake

^b^*p* value not corrected for multiple testing

^c^*p* value corrected for multiple testing, which was performed for 56 *p* values with a significance threshold of 0.05

^d^Adjusted for sex, HLA genotype, family history of diabetes of any type, total energy intake, and age at first seroconversion

The associations between vitamin C, D and E intakes and ICA plus biochemical IA were similar in the sensitivity analysis in which the 3 month age point was excluded (Table 3). Interaction with time was observed for the association of several vitamins and the risk of ICA plus biochemical IA (ESM Fig. 1). The associations for vitamins C, D and E were similar in most time-varying models compared with original models. However, based on the time-varying model, higher niacin intake may be associated with an increased risk of ICA plus biochemical IA at an early age but not after 2 years of age (ESM Fig. 1).

**Table 3:** Sensitivity analyses: child’s intake of A, B, C, D and E vitamins and the risk of IA at the age of 6 months to 6 years (ICA plus biochemical IA cohort)

	HR (95% CrI) (*n*=5143 participants, *n*=231)	*p* value
Vitamin A (10 µg/MJ)	0.97 (0.92, 1.01)	0.158
Retinol (10 µg/MJ)	0.96 (0.89, 1.02)	0.216
β-Carotene (100 µg/MJ)	0.93 (0.85, 1.01)	0.090
Carotenoids (100 µg/MJ)	0.95 (0.89, 1.01)	0.077
B vitamins		
Thiamine (10 µg/MJ)	0.98 (0.94, 1.00)	0.091
Riboflavin (10 µg/MJ)	0.99 (0.98, 1.01)	0.398
Niacin (1 mg/MJ)	0.94 (0.72, 1.20)	0.642
Pyridoxine (10 µg/MJ)	0.99 (0.96, 1.01)	0.298
Folate (10 µg/MJ)	0.87 (0.61, 1.14)	0.402
Vitamin B_12_ (0.1 µg/MJ)	0.98 (0.89, 1.05)	0.695
Vitamin C (10 mg/MJ)	0.68 (0.52, 0.88)	0.003
Vitamin D (1 µg/MJ)	0.82 (0.68, 0.99)	0.034
Vitamin E (0.1 mg/MJ)	0.95 (0.91, 0.99)	0.008
γ-Tocopherol (0.1 mg/MJ)	0.97 (0.92, 1.02)	0.319

Values are HR and CrIs from joint models per 1 unit increase in intake, e.g. per 10 µg/MJ increase for vitamin A. Adjusted for sex, HLA genotype, family history of diabetes of any type, and total energy intake

Sex or HLA genotype did not modify the associations between vitamin intakes and the risk of ICA plus biochemical IA or type 1 diabetes at the significance level of 0.05. The associations between vitamin intakes and outcomes were similar in the sensitivity analyses in which we excluded 590 children who were avoiding vegetables and fruits, 551 who were avoiding dairy or 405 who were avoiding cereals due to suspected food allergies (data not shown). Vitamin supplement use was not associated with the risk of ICA plus biochemical IA (data not shown).

Minor differences in vitamin intakes at the age of 6 months were observed by sex or family history of diabetes (ESM Table 3). The intake of retinol was higher in children whose mothers were more highly educated, whereas the intake of carotenoids was higher in children whose mothers were less highly educated. The intakes at the age of 6 months for all vitamins other than vitamin A were higher in children of less highly educated mothers compared with those of more highly educated mothers (ESM Table 3).

At the age of 2 years, the intakes of carotenoids, thiamine, niacin, folate, vitamins C, D and E, and γ-tocopherol were higher in children with more highly educated mothers compared to those with less highly educated mothers (ESM Table 4). At the age of 6 months, almost all vitamin intakes were higher for non-breastfed children than for breastfed children, reflecting the lower vitamin content of breastmilk compared with infant formulas and solid foods (ESM Table 5).

## Discussion

In this prospective birth cohort study that included children genetically at risk for type 1 diabetes, the intakes of vitamins C and E were consistently associated with decreased risk of ICA plus biochemical IA and multiple biochemical IA. These associations remained after correction for multiple testing.

### Strengths and limitations of the study

The strengths of our study are the large study population, regular assessment of autoantibodies, long follow-up, longitudinally assessed diet, and accurately measured food consumption and dietary supplement use. The food records were validated against serum concentrations of fatty acids [39] and carotenoids [40], and were shown to perform well. Furthermore, our food composition database considers the loss of vitamins in foods during cooking (except vitamin D and E). Our data also include a broad variety of covariates. Taking into account maternal education and breastfeeding as potential confounders was important as both are associated with disease outcomes and diet and also with each other [41]. Another strength is the use of joint models that analyse all available food records for each child, and therefore decrease bias related to missing data. Some limitations need to be acknowledged. Our study included children up to 6 years of age who live in Finland and are genetically at risk for type 1 diabetes. Thus, we cannot generalise the results to all children or to children who develop IA or type 1 diabetes after 6 years of age. The short follow-up also limits the number of children who developed type 1 diabetes, resulting in limited statistical power. Use of short-term food records may cause some measurement error, e.g. they may not detect rarely consumed foods, and thus may have some effect on the variation in vitamin intakes. However, use of repeated measurements and joint modelling help to manage this issue. Furthermore, young children have more stable diets than older children and thus the error related to short-term assessment is smaller [42]. Our study did not include plasma or serum vitamin measurements. Additionally, our study did not include measurements of breastmilk vitamin content from participating mothers. We were unable to measure or estimate levels of B vitamins produced by human gut microbes, genetic factors influencing metabolism of vitamins, sun exposure or deficient absorption of vitamins. Furthermore, we have previously observed that the results obtained when using joint models are not always in line with the results obtained when using Cox proportional hazard regression [43]. Finally, as our study is observational, no conclusions about causality can be made.

### Comparison with other studies

To the best of our knowledge, our study is one of the few to prospectively explore the association between longitudinal intake of a broad variety of vitamins and the risk of IA, type 1 diabetes or progression to type 1 diabetes. Most previous studies are retrospective case–control studies [2, 44]. Although our observations on the risk of type 1 diabetes did not hold for multiple testing, they were in line with our observed associations with IA.

Our novel observations relating to vitamin A suggest that retinol intake in particular is associated with decreased risk of IA. This observation is in line with the results of case–control and cross-sectional studies assessing plasma or serum vitamin A status [9, 10]. Vitamin A has been observed to play a role in pancreas development, glucose homeostasis and pancreatic innate immunity [45]. Furthermore, vitamin A and retinoic acid may enhance immune tolerance and thus protect beta cells against autoimmune islet inflammation [46]. However, our observed association between retinol and the risk of IA was not significant when the 3 month age point was excluded from the analysis. Breastmilk was the main source of retinol during the first year of life, but breastfeeding did not confound the association between retinol intake and the risk of IA. Therefore, breastfeeding may not explain the observed associations. Our observation on retinol is intriguing but the exclusion of the 3 month age point increases the uncertainty of our results and they should therefore be interpreted with caution.

Our null findings regarding B vitamins and type 1 diabetes-related outcomes are partly in line with observations from the TEDDY and DAISY studies [19, 20]. However, we were not able to replicate the association of niacin, pyridoxine or vitamin B_12_ intake with decreased risk of multiple biochemical IA observed in the TEDDY study, nor the association between metabolite-related nutrient pattern of riboflavin, niacin and vitamin B_12_ with decreased risk of progression to type 1 diabetes observed in the DAISY study.

Previous prospective studies on vitamin C and type 1 diabetes have assessed plasma vitamin status. In our previous study in the TEDDY cohort, high plasma ascorbic acid status in childhood was associated with decreased risk of IA [12], which is in line with the current findings. Type 1 diabetes development may result from intracellular formation of reactive oxygen species in beta cells [47]. Vitamin C functions as antioxidant by scavenging oxygen radicals. Antioxidant enzyme activity is low in beta cells, and thus vitamin C may be an essential dietary antioxidant for beta cells [47]. In our previous study within the DIPP study, high consumption of cruciferous vegetables was associate with decreased risk of IA and consumption of berries was associated with decreased risk of type 1 diabetes [48]. These associations may be partly explained by the role of vitamin C.

In our study, high intake of vitamin D was associated with decreased risk of IA, but not when corrected for multiple testing. A birth cohort study in Northern Finland that enrolled women who gave birth in 1966 observed that supplementation with 50 µg/day of vitamin D (a recommendation during the 1960s when the studied children were born) was associated with decreased incidence of type 1 diabetes [8], while the later DAISY cohort study observed no association between dietary intake of vitamin D and either IA or progression to type 1 diabetes [49]. In the earlier DIPP study, no association between the serum 25-hydroxyvitamin D level and the development of IA or type 1 diabetes was found [6, 7], whereas, in the TEDDY study, a combination of *HLA-DR4* genotype, vitamin D receptor variant and high serum vitamin D level was associated with decreased risk of IA [5]. Thus, further prospective studies combining both vitamin D intake and status are warranted.

Our study is the first to observe an association between vitamin E intake and decreased risk of IA and type 1 diabetes. This finding complements those of earlier studies that observed a borderline protective association between low plasma α-tocopherol concentration and increased risk of type 1 diabetes [14, 15]. Vitamin E is a fat-soluble antioxidant that is known to protect cell membranes from oxidation, enhance the immune system and decrease insulin resistance [50]. A recent retrospective incident case–control study suggested that high vitamin E intake may protect against latent autoimmune diabetes in adults (LADA), particularly in participants with high autoantibody levels [51]. Our novel observation suggests a similar association for type 1 diabetes in children but requires confirmation in further studies.

At 6 months, children of less highly educated mothers compared with those of more highly educated mothers had higher intakes of all vitamins except vitamin A. This is due to the lower frequency of breastfeeding among less highly educated mothers and the lower content of most vitamins in breastmilk compared with infant formula and other complementary foods [52]. At 2 years, children of more highly educated mothers had higher intakes of all vitamins except vitamin A, retinol, riboflavin, pyridoxine and vitamin B_12_. Children with more highly educated mothers are likely to eat more health consciously than children with less highly educated mothers [53]. Furthermore, we observed in a previous DIPP study that higher maternal education level was associated with decreased risk of IA and increased risk of progression to type 1 diabetes [41]. However, in the current study, the adjustment for maternal education did not change the association between vitamin intakes and the risk of IA or type 1 diabetes.

Breastmilk and dairy products were the main sources of all vitamins, except for carotenoids, before the age of 12 months. Meats represent the main sources of A and B vitamins, while juices were the main source for vitamin C and fats were the main source for vitamin E from the second year of life onwards. In Finland, milk, other dairy products and fat spreads have been fortified with vitamin D since 2003, which explains the significance of dairy products as the second primary source of vitamin D in our study, after supplements. Diet was the main source of vitamins, and vitamin supplement use was not associated with the risk of IA. This is in line with the general recommendation that a diverse diet that provides a broad variety of vitamins is a good foundation for healthy childhood.

### Conclusion

Higher intakes of vitamins C and E were associated with decreased risk of IA in children who are genetically at risk for type 1 diabetes. suggesting the potential for prevention by dietary means. Our observed associations are novel and thus need to be confirmed in further mechanistic and aetiological studies.

## Supplementary Information

Below is the link to the electronic supplementary material.

ESM (PDF 575 KB)

## Data Availability

The data described in this manuscript and analysis codes may be made available upon request subject to ethical and legislative restrictions.

## Abbreviations

CrI: Credible interval
DIPP: Finnish Type 1 Diabetes Prediction and Prevention Study
IA: Islet autoimmunity
