# Supplementary material for: Dietary intake of vitamins A, B, C, D and E and risk of islet autoimmunity and type 1 diabetes in genetically at-risk children: a prospective study from the DIPP birth cohort
Source: Diabetologia. 2025 Dec 19;69(4):917–29. doi: 10.1007/s00125-025-06635-9 (PMC12957001; doi:10.1007/s00125-025-06635-9)
Supplement: Supplementary file 1 — ESM (PDF 575 KB) [file 125_2025_6635_MOESM1_ESM.pdf]

*ESM Results: intake and sources of vitamins*

Dairy products represent the important food source of all vitamins studied among children in both up to 1-year of age and after the first year of life (ESM Tables 1-2). In children up to 1 year of age, dairy products were the primary source of vitamins due to use of infant formulas. Breastmilk was also a prominent source of most vitamins with exception of vitamin D, ß-carotene, and carotenoids.

Red meat was one of the main food sources of vitamins A, retinol, thiamine, niacin, pyridoxin, and vitamin $B_{12}$ (ESM Tables 1-2). Cereals were the important sources of vitamins A, retinol, thiamine, niacin, and folate. The potato and potato products were common food source of thiamine and folate. Fruit, berries, juices, potato, and potato products were the most common food sources of vitamin C, covering more than 50% of daily intake in children aged 2 to 6 years.

Root vegetables represent the most important food sources of vitamin A, ß-carotene, and carotenoids (ESM Table 1). Margarines and oils were major sources of vitamin A, retinol, and vitamin E.

Children aged 2 to 6 years received a significant proportion of some vitamins from dietary supplements, e.g. the thiamine (11%), pyridoxine (10%) and vitamin E (14%). Over 60% of the daily intake of vitamin D came from dietary supplements at ages 3, 6 and 12 months, and 42% at ages 2 to 6 years.

ESM Table 1 The mean dietary intake of A, C, D, and E vitamins from foods and dietary supplements and proportion of intake by food groups in DIPP Study participants

| | Vitamin A | | Retinol | | ß-carotene | | Carotenoids | | Vitamin C | | Vitamin D | | Vitamin E | |
|---|---|---|---|---|---|---|---|---|---|---|---|---|---|---|
| Age groups | ≤12 months (*n*=5588) | 2-6 years (*n*=4173) | ≤12 months (*n*=5588) | 2-6 years (*n*=4173) | ≤12 months (*n*=5588) | 2-6 years (*n*=4173) | ≤12 months (*n*=5588) | 2-6 years (*n*=4173) | ≤12 months (*n*=5588) | 2-6 years (*n*=4173) | ≤12 months (*n*=5588) | 2-6 years (*n*=4173) | ≤12 months (*n*=5588) | 2-6 years (*n*=4173) |
| Mean intake/day | 1317 µg | 1648 µg | 953 µg | 1206 µg | 3616 µg | 4529 µg | 4388 µg | 5323 µg | 182 mg | 216 mg | 27.5 µg | 20.3 µg | 10.8 mg | 15.8 mg |
| % of intake by food groups | | | | | | | | | | | | | | |
| Breastmilk | 40.9 | [a] | 54.3 | [a] | 6.57 | [a] | 5.78 | [a] | 32.7 | [a] | 1.26 | [a] | 32.0 | [a] |
| Dairy products[b] | 28.7 | 20.2 | 39.1 | 26.2 | 1.59 | 4.52 | 1.32 | 3.91 | 27.5 | 11.4 | 33.6 | 31.4 | 37.2 | 6.87 |
| Meat and meat products | 2.38 | 25.5 | 3.25 | 34.6 | 0.16 | 0.78 | 0.13 | 0.67 | 0.02 | 0.31 | 0.56 | 3.59 | 1.92 | 7.30 |
| Red meat | 2.05 | 24.6 | 2.79 | 33.4 | 0.15 | 0.69 | 0.13 | 0.59 | 0.02 | 0.31 | 0.31 | 1.56 | 1.14 | 3.14 |
| Fish and fish products | 0.05 | 0.33 | 0.08 | 0.45 | <0.10 | <0.10 | <0.10 | <0.10 | <0.10 | <0.10 | 1.00 | 8.19 | 0.61 | 2.70 |
| Egg | 0.42 | 4.11 | 0.52 | 5.60 | <0.10 | 0.07 | <0.10 | 0.06 | [a] | [a] | 0.16 | 2.72 | 0.38 | 3.26 |
| Cereals | 0.04 | 0.40 | 0.05 | 0.44 | 0.04 | 0.34 | 0.03 | 0.31 | <0.10 | <0.10 | <0.10 | 0.09 | 2.98 | 9.11 |
| Fruits | 0.84 | 1.05 | <0.10 | <0.10 | 3.12 | 2.73 | 3.05 | 3.91 | 8.26 | 14.3 | <0.10 | <0.10 | 3.50 | 4.03 |
| Berries | 0.61 | 0.18 | <0.10 | <0.10 | 2.67 | 0.80 | 2.21 | 0.70 | 15.5 | 12.1 | [a] | [a] | 3.45 | 3.84 |
| Juices | 0.02 | 0.65 | <0.10 | <0.10 | 0.08 | 2.36 | 0.08 | 2.42 | 1.64 | 25.5 | [a] | [a] | 0.15 | 2.86 |
| Vegetables | 24.0 | 20.3 | <0.10 | <0.10 | 85.3 | 74.1 | 87.0 | 75.6 | 8.78 | 16.9 | <0.10 | 0.10 | 2.45 | 4.85 |
| Root vegetables | 23.2 | 17.2 | <0.10 | <0.10 | 81.8 | 61.5 | 83.9 | 64.0 | 1.84 | 2.88 | [a] | [a] | 1.42 | 0.99 |
| Potato and potato products | 0.05 | 0.09 | <0.10 | <0.10 | 0.20 | 0.37 | 0.17 | 0.32 | 5.36 | 10.3 | [a] | [a] | 0.49 | 3.23 |
| Fats and oils | 1.42 | 18.5 | 1.92 | 24.9 | 0.12 | 1.23 | 0.10 | 1.05 | <0.10 | <0.10 | 0.43 | 11.2 | 14.4 | 35.4 |
| Margarines and oils | 0.73 | 11.3 | 1.01 | 15.4 | <0.10 | 0.03 | <0.10 | 0.03 | <0.10 | <0.10 | 0.31 | 8.83 | 13.7 | 26.7 |
| Other foods | 0.05 | 3.52 | 0.02 | 0.80 | 0.16 | 12.5[c] | 0.13 | 11.0[c] | <0.10 | 0.52 | <0.10 | 0.12 | 0.05 | 2.85 |
| Dietary supplements | 0.53 | 5.15 | 0.73 | 7.00 | [a] | 0.11 | [a] | 0.09 | 0.26 | 8.71 | 63.0 | 42.5 | 0.49 | 13.6 |
| Total | 100 | 100 | 100 | 100 | 100 | 100 | 100 | 100 | 100 | 100 | 100 | 100 | 100 | 100 |

[a] Not a significant source of vitamin in question at this age group
[b] Includes infant formulas
[c] High proportional intake of ß-carotene (12.5%) and carotenoids (11%) from other foods are explained by the consumption of ketchup

ESM Table 2 The mean dietary intake of B vitamins from foods and dietary supplements and the proportion of intake by food groups in DIPP Study participants.

| | Thiamine | | Riboflavin | | Niacin | | Pyridoxine | | Folate | | Vitamin $B_{12}$ | |
|---|---|---|---|---|---|---|---|---|---|---|---|---|
| Age groups | ≤12 months (*n*=5588) | 2-6 years (*n*=4173) | ≤12 months (*n*=5588) | 2-6 years (*n*=4173) | ≤12 months (*n*=5588) | 2-6 years (*n*=4173) | ≤12 months (*n*=5588) | 2-6 years (*n*=4173) | ≤12 months (*n*=5588) | 2-6 years (*n*=4173) | ≤12 months (*n*=5588) | 2-6 years (*n*=4173) |
| Mean intake/day | 1.27 mg | 2.97 mg | 2.39 mg | 5.21 mg | 20.6 mg | 58.5 mg | 1.37 mg | 4.19 mg | 211 µg | 455 µg | 3.92 µg | 13.1 µg |
| % of intake by food groups | | | | | | | | | | | | |
| Breastmilk | 15.6 | [a] | 16.5 | [a] | 18.9 | [a] | 7.21 | [a] | 23.5 | [a] | 12.6 | [a] |
| Dairy products[b] | 38.6 | 21.6 | 70.7 | 66.3 | 39.5 | 29.6 | 43.9 | 21.5 | 34.9 | 19.7 | 66.4 | 55.1 |
| Meat and meat products | 11.1 | 19.8 | 3.74 | 9.20 | 16.9 | 27.0 | 13.6 | 18.9 | 1.94 | 9.31 | 15.6 | 31.1 |
| Red meat | 9.70 | 11.9 | 2.80 | 5.24 | 12.1 | 14.7 | 9.88 | 10.9 | 1.25 | 7.49 | 12.9 | 23.7 |
| Fish and fish products | 0.21 | 0.65 | 0.16 | 0.47 | 1.85 | 4.18 | 1.20 | 2.46 | 0.22 | 0.62 | 3.67 | 6.88 |
| Egg | 0.19 | 1.05 | 0.34 | 1.92 | 0.37 | 1.65 | 0.21 | 0.86 | 0.44 | 2.98 | 1.38 | 5.16 |
| Cereals | 9.93 | 18.0 | 1.90 | 5.08 | 11.0 | 19.3 | 6.56 | 9.27 | 9.13 | 16.5 | <0.10 | <0.10 |
| Fruits | 1.91 | 2.44 | 1.76 | 1.56 | 3.08 | 2.48 | 9.57 | 8.69 | 2.58 | 3.89 | <0.10 | <0.10 |
| Berries | 0.55 | 0.59 | 0.61 | 0.68 | 0.41 | 0.41 | 0.97 | 0.72 | 2.74 | 2.74 | [a] | [a] |
| Juices | 0.42 | 2.99 | 0.12 | 0.93 | 0.08 | 0.67 | 1.96 | 14.0 | 0.44 | 5.00 | <0.10 | <0.10 |
| Vegetables | 5.45 | 4.97 | 2.46 | 2.67 | 3.66 | 3.17 | 5.13 | 4.45 | 12.0 | 10.8 | <0.10 | <0.10 |
| Root vegetables | 2.47 | 1.14 | 1.30 | 0.67 | 1.39 | 0.69 | 1.31 | 0.63 | 4.10 | 2.54 | [a] | [a] |
| Potato and potato products | 15.5 | 15.0 | 1.22 | 1.36 | 3.66 | 2.76 | 9.00 | 7.71 | 10.4 | 10.7 | [a] | [a] |
| Fats and oils | <0.10 | <0.10 | <0.10 | <0.10 | <0.10 | <0.10 | <0.10 | <0.10 | <0.10 | 0.13 | <0.10 | 0.10 |
| Margarines and oils | <0.10 | <0.10 | <0.10 | <0.10 | <0.10 | <0.10 | <0.10 | <0.10 | <0.10 | <0.1 | <0.10 | <0.10 |
| Other foods | 0.12 | 1.83 | 0.23 | 2.71 | 0.22 | 2.11 | 0.12 | 1.17 | 1.44 | 11.1c[c] | 0.08 | 0.11 |
| Dietary supplements | 0.41 | 11.1 | 0.24 | 6.99 | 0.26 | 6.55 | 0.53 | 10.2 | 0.27 | 6.51 | 0.25 | 1.45 |
| Total | 100 | 100 | 100 | 100 | 100 | 100 | 100 | 100 | 100 | 100 | 100 | 100 |

[a] Not a significant source of vitamin in question at this age group
[b] Includes infant formulas
[c] High proportional intake of folate from other foods (11%) is explained by the consumption of yeast

ESM Table 3 Energy adjusted mean (±SD) vitamin intakes by background variables at 6 months of age in DIPP Study participants.

| | Sex | | | HLA conferred risk | | | Family history of diabetes | | | Maternal education | | | | |
|---|---|---|---|---|---|---|---|---|---|---|---|---|---|---|
| Variable | Male | Female | *p* value[a] | Moderate | High | *p* value[a] | No | Yes | *p* value[a] | None | Vocational school or course | Secondary vocational education | University studies or degree | *p* value[a] |
| Vitamin A, µg/MJ | 202 (56.8) | 205 (57.2) | 0.040 | 203 (56.6) | 204 (58.7) | 0.674 | 203 (56.1) | 204 (64.8) | 0.775 | 198 (523) | 201 (54.4) | 204 (60.8) | 205 (50.7) | 0.095 |
| Retinol, µg/MJ | 132 (34.3) | 134 (35.3) | 0.046 | 133 (35.1) | 132 (34.0) | 0.482 | 133 (34.7) | 134 (34.9) | 0.571 | 126 (33.9) | 132 (35.5) | 132 (34.2) | 139 (34.1) | <0.001 |
| ß-carotene, µg/MJ | 639 (520) | 653 (517) | 0.372 | 643 (510) | 657 (553) | 0.464 | 642 (510) | 640 (597) | 0.945 | 659 (489) | 633 (488) | 639 (552) | 607 (468) | 0.031 |
| Carotenoids, µg/MJ | 842 (701) | 858 (693) | 0.409 | 845 (685) | 867 (744) | 0.425 | 845 (686) | 844 (800) | 0.983 | 871 (656) | 835 (656) | 871 (742) | 796 (630) | 0.028 |
| Thiamine, µg/MJ | 165 (57.7) | 163 (57.4) | 0.168 | 164 (57.7) | 166 (57.2) | 0.217 | 163 (57.6) | 171 (58.0) | 0.028 | 187 (52.3) | 174 (57.4) | 164 (56.5) | 144 (55.9) | <0.001 |
| Riboflavin, µg/MJ | 265 (127) | 264 (126) | 0.801 | 264 (126) | 267 (128) | 0.525 | 262 (127) | 281 (126) | 0.018 | 315 (126) | 290 (128) | 261 (125) | 226 (117) | <0.001 |
| Niacin, mg/MJ | 2.22 (0.58) | 2.21 (0.58) | 0.468 | 2.21 (0.58) | 2.24 (0.57) | 0.136 | 2.20 (0.58) | 2.28 (0.56) | 0.047 | 2.40 (0.53) | 2.31 (0.57) | 2.22 (0.59) | 2.05 (0.55) | <0.001 |
| Pyridoxine, µg/MJ | 193 (87.8) | 189 (87.2) | 0.137 | 191 (88.3) | 193 (84.5) | 0.526 | 190 (88.0) | 202 (88.3) | 0.018 | 221 (78.0) | 207 (86.3) | 192 (85.7) | 162 (88.5) | <0.001 |
| Folate, µg/MJ | 27.3 (6.39) | 27.2 (6.31) | 0.544 | 27.1 (6.36) | 27.4 (6.30) | 0.416 | 27.2 (6.36) | 27.8 (6.72) | 0.100 | 28.3 (5.83) | 27.8 (6.12) | 27.4 (6.50) | 25.9 (6.31) | <0.001 |
| Vitamin $B_{12}$, µg/MJ | 0.38 (0.19) | 0.34 (0.18) | 0.232 | 0.37 (0.18) | 0.38 (0.18) | 0.236 | 0.37 (0.18) | 0.41 (0.19) | 0.003 | 0.45 (0.17) | 0.41 (0.18) | 0.37 (0.18) | 0.31 (0.17) | <0.001 |
| Vitamin C, mg/MJ | 29.2 (6.85) | 28.9 (6.50) | 0.071 | 29.1 (6.73) | 29.1 (6.51) | 0.800 | 29.0 (6.56) | 29.3 (7.99) | 0.476 | 29.6 (7.02) | 29.3 (6.78) | 29.2 (6.55) | 28.2 (6.53) | <0.001 |
| Vitamin D, µg/MJ | 4.12 (1.50) | 4.32 (1.64) | <0.001 | 4.19 (1.58) | 4.27 (1.51) | 0.160 | 4.21 (1.58) | 4.19 (1.41) | 0.851 | 4.34 (1.54) | 4.37 (1.62) | 4.25 (1.58) | 3.93 (1.44) | <0.001 |
| Vitamin E, mg/MJ | 1.67 (0.47) | 1.66 (0.46) | 0.707 | 1.66 (0.46) | 1.69 (0.48) | 0.075 | 1.66 (0.46) | 1.71 (0.54) | 0.089 | 1.79 (0.45) | 1.73 (0.47) | 1.67 (0.48) | 1.54 (0.42) | <0.001 |
| γ-tocopherol, mg/MJ | 0.84 (0.54) | 0.84 (0.52) | 0.885 | 0.83 (0.53) | 0.86 (0.53) | 0.106 | 0.84 (0.53) | 0.90 (0.55) | 0.073 | 1.00 (0.49) | 0.92 (0.51) | 0.84 (0.54) | 0.70 (0.50) | <0.001 |

[a] *p* values for difference between groups from one-factor ANOVA or unpaired t-test.

ESM Table 4 Energy adjusted mean (±SD) vitamin intakes by background variables at 2 years of age in the DIPP Study participants.

| | Sex | | | HLA conferred risk | | | Family history of diabetes | | | Maternal education | | | | |
|---|---|---|---|---|---|---|---|---|---|---|---|---|---|---|
| Variable | Male | Female | *p* value[a] | Moderate | High | *p* value[a] | No | Yes | *p* value[a] | None | Vocational school or course | Secondary vocational education | University studies or degree | *p* value[a] |
| Vitamin A, µg/MJ | 102 (109) | 102 (107) | 0.920 | 102 (109) | 101 (106) | 0.730 | 102 (109) | 94.9 (103) | 0.284 | 103 (107) | 99.6 (104) | 103 (106) | 104 (120) | 0.204 |
| Retinol, µg/MJ | 73.8 (106) | 73.2 (103) | 0.789 | 73.6 (105) | 73.1 (102) | 0.881 | 74.0 (105) | 66.4 (92.6) | 0.235 | 79.4 (106) | 72.3 (99.1) | 73.9 (102) | 73.9 (118) | 0.567 |
| ß-carotene, µg/MJ | 273 (247) | 276 (288) | 0.540 | 276 (275) | 266 (232) | 0.319 | 274 (268) | 274 (254) | 0.924 | 231 (160) | 264 (288) | 283 (258) | 292 (258) | <0.001 |
| Carotenoids, µg/MJ | 340 (318) | 344 (377) | 0.556 | 344 (358) | 333 (300) | 0.413 | 342 (348) | 343 (335) | 0.999 | 288 (205) | 330 (380) | 353 (331) | 363 (331) | <0.001 |
| Thiamine, µg/MJ | 166 (57.9) | 167 (55.2) | 0.654 | 167 (57.8) | 165 (51.6) | 0.356 | 166 (56.9) | 169 (54.6) | 0.461 | 165 (60.3) | 165 (57.0) | 164 (53.5) | 173 (61.5) | 0.005 |
| Riboflavin, µg/MJ | 312 (101) | 319 (95.5) | 0.039 | 316 (99.0) | 314 (97.1) | 0.597 | 316 (98.6) | 316 (98.4) | 0.965 | 315 (108) | 318 (103) | 312 (95.7) | 319 (96.6) | 0.285 |
| Niacin, mg/MJ | 3.27 (0.76) | 3.26 (0.72) | 0.781 | 3.27 (0.75) | 3.24 (0.72) | 0.266 | 3.27 (0.75) | 3.23 (0.70) | 0.388 | 3.22 (0.81) | 3.22 (0.76) | 3.24 (0.71) | 3.40 (0.76) | <0.001 |
| Pyridoxine, µg/MJ | 264 (93.3) | 257 (77.6) | 0.009 | 261 (88.8) | 257 (75.1) | 0.180 | 261 (86.5) | 265 (88.3) | 0.487 | 253 (86.4) | 257 (85.8) | 260 (87.8) | 267 (84.6) | 0.049 |
| Folate, µg/MJ | 24.2 (7.86) | 24.0 (8.13) | 0.490 | 24.2 (8.03) | 23.9 (7.83) | 0.582 | 24.1 (8.02) | 23.8 (7.40) | 0.452 | 23.4 (6.68) | 23.5 (7.73) | 24.4 (7.58) | 25.0 (9.13) | <0.001 |
| Vitamin $B_{12}$, µg/MJ | 0.79 (0.43) | 0.80 (0.39) | 0.756 | 0.79 (0.41) | 0.79 (0.40) | 0.530 | 0.79 (0.41) | 0.76 (0.40) | 0.184 | 0.78 (0.41) | 0.78 (0.38) | 0.80 (0.42) | 0.81 (0.45) | 0.603 |
| Vitamin C, mg/MJ | 13.2 (8.02) | 12.7 (7.39) | 0.206 | 12.9 (7.75) | 12.9 (11.0) | 0.821 | 12.9 (7.75) | 13.3 (7.51) | 0.423 | 12.2 (7.50) | 12.3 (7.39) | 13.3 (7.49) | 13.8 (8.58) | <0.001 |
| Vitamin D, µg/MJ | 1.76 (1.05) | 1.83 (1.10) | 0.016 | 1.81 (1.08) | 1.73 (1.03) | 0.029 | 1.80 (1.08) | 1.80 (1.06) | 0.880 | 1.69 (1.03) | 1.73 (1.10) | 1.84 (1.07) | 1.88 (1.04) | <0.001 |
| Vitamin E, mg/MJ | 0.91 (0.57) | 0.89 (0.43) | 0.175 | 0.91 (0.54) | 0.87 (0.37) | 0.056 | 0.90 (0.52) | 0.90 (0.44) | 0.949 | 0.87 (0.51) | 0.87 (0.57) | 0.92 (0.45) | 0.93 (0.47) | <0.001 |
| γ-tocopherol, mg/MJ | 0.60 (0.34) | 0.60 (0.33) | 0.420 | 0.60 (0.34) | 0.60 (0.32) | 0.613 | 0.60 (0.34) | 0.60 (0.29) | 0.492 | 0.60 (0.37) | 0.57 (0.32) | 0.61 (0.34) | 0.62 (0.34) | 0.036 |

[a] *p* values for difference between groups from one-factor ANOVA or unpaired *t* test.

ESM Table 5 Energy adjusted mean (±SD) vitamin intakes at the age of 6 months and 2 years in children breastfed vs. not at the age of 6 months in the DIPP Study participants.

| Age point | Vitamin intake at 6 months | | | Vitamin intake at 2 years | | |
|---|---|---|---|---|---|---|
| | Breastfed at 6 months of age | | | Breastfed at 6 months of age | | |
| | No (*n*=1987) | Yes (*n*=3011) | *p* value[a] | No (*n*=1307) | Yes (*n*=2334) | *p* value[a] |
| Vitamin A, µg/MJ | 198 (57.0) | 207 (56.7) | <0.001 | 107 (125) | 98.4 (95.5) | 0.022 |
| Retinol, µg/MJ | 129 (33.5) | 135 (35.4) | <0.001 | 78.8 (120) | 70 (93.2) | 0.013 |
| ß-carotene, µg/MJ | 628 (492) | 657 (535) | 0.054 | 270 (322) | 275 (227) | 0.574 |
| Carotenoids, µg/MJ | 832 (662) | 860 (719) | 0.171 | 338 (424) | 343 (291) | 0.716 |
| Thiamine, µg/MJ | 214 (33.0) | 133 (47.0) | <0.001 | 168 (57.8) | 166 (55.9) | 0.312 |
| Riboflavin, µg/MJ | 383 (90.5) | 190 (82.3) | <0.001 | 325 (104) | 310 (94.8) | <0.001 |
| Niacin, mg/MJ | 2.68 (0.45) | 1.93 (0.45) | <0.001 | 3.27 (0.76) | 3.26 (0.73) | 0.816 |
| Pyridoxine, µg/MJ | 265 (43.6) | 145 (76.1) | <0.001 | 262 (95.8) | 260 (80.6) | 0.597 |
| Folate, µg/MJ | 31.7 (5.05) | 24.4 (5.41) | <0.001 | 23.9 (8.19) | 24.2 (7.88) | 0.196 |
| Vitamin $B_{12}$, µg/MJ | 0.55 (0.11) | 0.26 (0.13) | <0.001 | 0.82 (0.44) | 0.77 (0.39) | 0.003 |
| Vitamin C, mg/MJ | 30.8 (6.59) | 28.0 (6.53) | <0.001 | 12.5 (7.38) | 13.1 (7.93) | 0.040 |
| Vitamin D, µg/MJ | 5.06 (1.18) | 3.68 (1.55) | <0.001 | 1.71 (1.11) | 1.84 (1.28) | 0.002 |
| Vitamin E, mg/MJ | 2.01 (0.41) | 1.45 (0.36) | <0.001 | 0.90 (0.60) | 0.90 (0.45) | 0.960 |
| γ-tocopherol, mg/MJ | 1.28 (0.41) | 0.57 (0.40) | <0.001 | 0.60 (0.35) | 0.60 (0.32) | 0.702 |

[a] *p* values for difference between groups from *t* test.

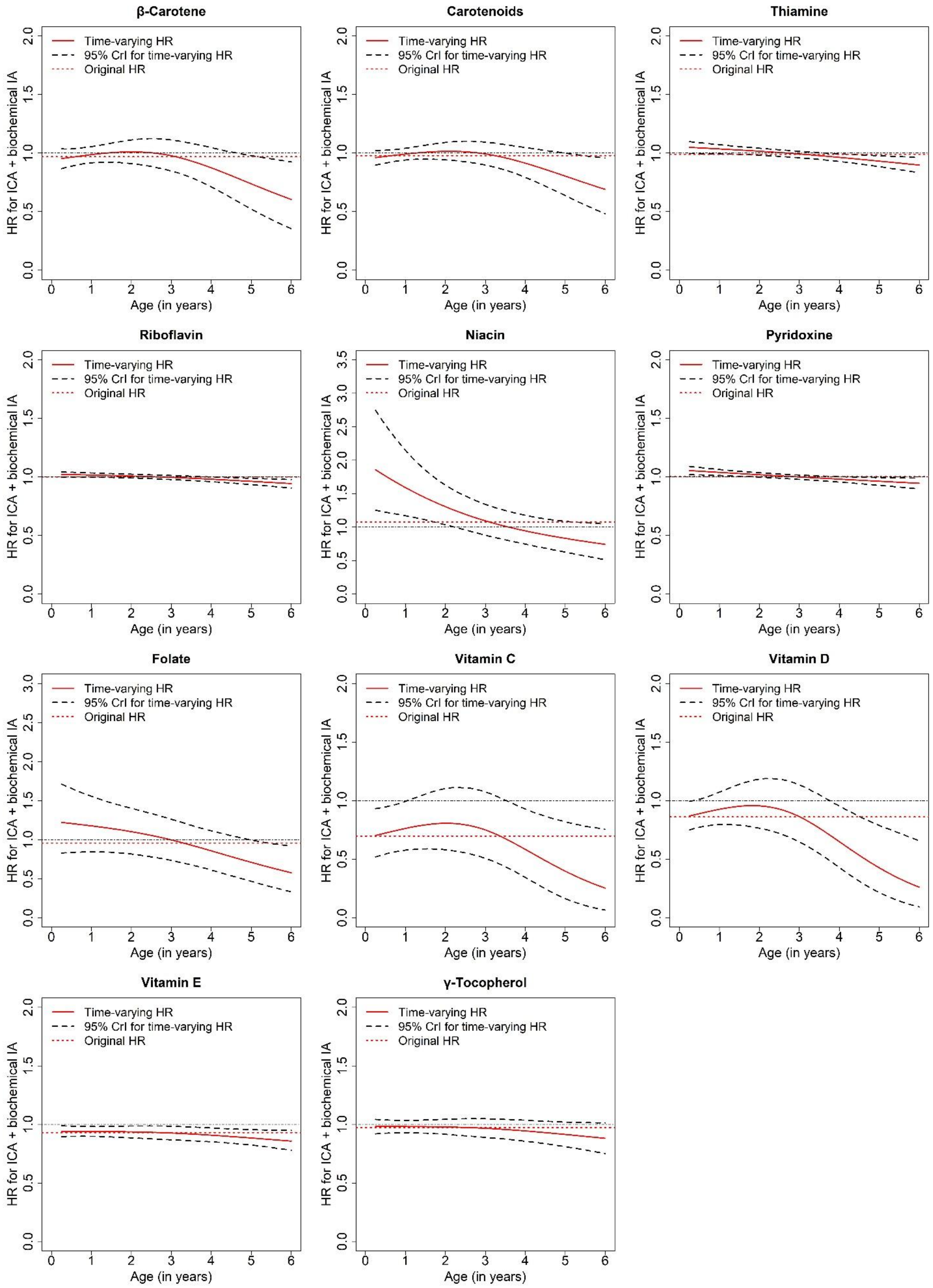


ESM Fig. 1 Time-varying associations with the risk of IA for the models with indication of time interaction. CrI, credible interval; IA, islet autoimmunity; ICA, islet cell autoantibodies

Red dashed lines represent the observed hazard ratio for the vitamin intake in question, while black dashed lines represent a hazard ratio of 1.00.
